# Silent but present: a case of Chagas disease-related megaoesophagus diagnosed in a non-endemic setting

**DOI:** 10.1093/jtm/taag014

**Published:** 2026-02-12

**Authors:** Virginia Donini, Anna Barbiero, Michele Spinicci, Fabio Marra, Francesco Vizzutti, Damiano Bisogni, Gian Maria Rossolini, Francesca Malentacchi, Lorenzo Zammarchi, Alessandro Bartoloni

**Affiliations:** Department of Experimental and Clinical Medicine, University of Florence, Largo Brambilla, 3 - 50134 Florence (FI), Italy; Department of Experimental and Clinical Medicine, University of Florence, Largo Brambilla, 3 - 50134 Florence (FI), Italy; Department of Experimental and Clinical Medicine, University of Florence, Largo Brambilla, 3 - 50134 Florence (FI), Italy; Infectious and Tropical Diseases Unit, Careggi University Hospital, Largo Brambilla, 3 - 50134 Florence (FI), Italy; Department of Experimental and Clinical Medicine, University of Florence, Largo Brambilla, 3 - 50134 Florence (FI), Italy; Internal Medicine and Hepatology, Careggi University Hospital, Largo Brambilla, 3 - 50134 Florence (FI), Italy; Department of Experimental and Clinical Medicine, University of Florence, Largo Brambilla, 3 - 50134 Florence (FI), Italy; Internal Medicine and Hepatology, Careggi University Hospital, Largo Brambilla, 3 - 50134 Florence (FI), Italy; Interventional Endoscopy, Careggi University Hospital, Largo Brambilla, 3 - 50134 Florence (FI), Italy; Department of Experimental and Clinical Medicine, University of Florence, Largo Brambilla, 3 - 50134 Florence (FI), Italy; Microbiology and Virology Unit, Careggi University Hospital, Largo Brambilla, 3 - 50134 Florence (FI), Italy; Microbiology and Virology Unit, Careggi University Hospital, Largo Brambilla, 3 - 50134 Florence (FI), Italy; Department of Experimental and Clinical Medicine, University of Florence, Largo Brambilla, 3 - 50134 Florence (FI), Italy; Infectious and Tropical Diseases Unit, Careggi University Hospital, Largo Brambilla, 3 - 50134 Florence (FI), Italy; Department of Experimental and Clinical Medicine, University of Florence, Largo Brambilla, 3 - 50134 Florence (FI), Italy; Infectious and Tropical Diseases Unit, Careggi University Hospital, Largo Brambilla, 3 - 50134 Florence (FI), Italy

## Abstract

Due to migration phenomena, Chagas disease (CD) is emerging in Europe, where CD-related gastrointestinal complications are rarely reported. We describe a case of CD-related megaoesophagus in a patient from El Salvador, in Italy since 14 years, highlighting the importance of considering complicated CD in patients at risk with suggestive symptoms.

## Introduction

Chagas disease (CD), a neglected tropical disease caused by the flagellate protozoa *Trypanosoma cruzi*, is primarily transmitted by Hemiptera insects, but can also be transmitted vertically, through blood and organ donation and, more rarely, the oral route.^1^

CD is endemic in continental Latin America and in some areas of the USA. In Europe, where the vector is not present, most cases are recorded in migrants from CD-endemic areas mostly living in Spain, Italy, Portugal, Sweden, Switzerland and the Netherlands.^2^

Following acute infection, if left untreated, CD progresses to a chronic phase with development of cardiac, digestive or neurological involvement in 30–40% of infected persons within 10–30 years, leading to increased morbidity and mortality.^3^

## Case description

A 63-year-old woman from El Salvador, living in Italy for 14 years and with no known comorbidities, presented with aggravating dysphagia and weight loss (17 kg) since September 2023. The patient’s mother had cardiological problems since an early age and died at 45 of sudden death.

**Figure 1:**
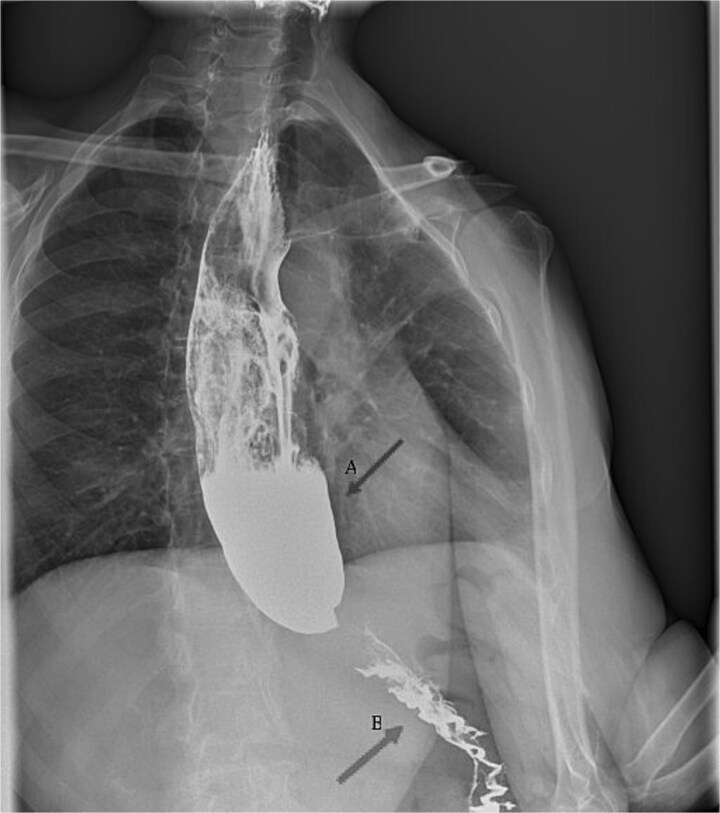
Barium swallow X-ray (January 2024) showing dilatation of the oesophagus (arrow A) and a tapered narrowing at the oesophagogastric junction (‘bird-beak sign’) (arrow B).

After multiple assessments, including a normal oesophagogastroduodenoscopy, a barium swallow X-ray in January 2024 demonstrated a dyscalasic megaoesophagus (Figure 1). The patient tested positive at the Western-Blot and chemiluminescent immunoassay (CMIA) serology for *T. cruzi*, confirming the diagnosis of chronic CD with gastrointestinal involvement. *T. cruzi* PCR on peripheral blood resulted negative.

In April 2024, the patient underwent a peroral endoscopic myotomy (POEM). After this procedure, she had a progressive improvement in her ability to feed with solid food and she gained 10 kg.

Cardiological evaluation and Holter electrocardiography were normal, as well as endoscopic and radiographic studies of the colon.

Following recovery after POEM, after a thorough discussion with the patient on the risks and benefits related to specific antiparasitic treatment, on 25 November 2024 she was started on benznidazole 150 mg bid, concluded after 60 days with no major adverse events. Approximately 10 days after initiating therapy, she developed a mildly itching cutaneous rash that did not require treatment interruption. At her most recent follow-up in May 2025, she remained in good clinical condition.

## Conclusion

This report underlines the importance of always suspecting CD in patients coming from endemic areas with gastrointestinal and/or cardiological symptoms; its prompt recognition would not only favour adequate clinical evaluation and management but would be essential to enable early treatment and limit chronic sequelae.

According to recent reviews, CD is estimated to affect over 100 000 individuals in Europe, with digestive involvement likely present in 9–21% of cases but rarely being reported.^4^ Estimates for migrant populations and country-specific prevalence data remain highly variable and likely underestimated^2^; in Spain, where the South American population is highly represented among immigrants, travellers and visiting friends and relatives, CD represented about one third of the diagnoses made on these groups.^5^ The adoption of spread and systematic screening policies in high-risk populations would be the key to reduce underdiagnosis rates of CD in Europe.

However, despite representing a relevant health issue for the affected population, Chagas disease-related gastrointestinal complications are rarely reported in the European setting, and in general this condition has received considerably less attention in terms of clinical and epidemiological aspects, compared to Chagas related-cardiac involvement.^6^

Untreated Chagas-related megaoesophagus is associated with a poor prognosis; however, timely POEM can reduce complications and achieve clinical success rates exceeding 90%; some studies have shown that POEM is an effective and safe treatment for Chagasic achalasia.^7^^,^^8^

Trypanocidal therapy is not strongly recommended for patients over 50 years of age with organ damage. However, in the described case, it was recommended to prevent cardiac involvement and/or reactivation during eventual future immunosuppression. Due to limited evidence regarding its efficacy in older patients with gastrointestinal involvement, treatment decisions should be individualized in such cases.^9^^,^^10^

## Data Availability

Data sharing is not applicable to this article as no new data were created or analysed in this study.
